# Voids healing and carbide refinement of cold rolled M50 bearing steel by electropulsing treatment

**DOI:** 10.1038/s41598-019-47919-6

**Published:** 2019-08-05

**Authors:** Feng Wang, Dongsheng Qian, Lin Hua, Huajie Mao, Lechun Xie

**Affiliations:** 1 School of Materials Science and Engineering, Wuhan University of Technology, Wuhan, 430070 China; 2 Hubei key Laboratory of Advanced Technology for Automotive Components, Wuhan, 430070 China; 3 School of Automotive Engineering, Wuhan University of Technology, Wuhan, 430070 China

**Keywords:** Metals and alloys, Design, synthesis and processing

## Abstract

The voids caused by the cold rolling (CR) quite deteriorates the final performance of M50 bearing steel. In this work, the effect of electropulsing treatment (EPT) on the voids has been investigated, finding that the nano-size voids around carbides have been extensively healed. Moreover, it is interesting to find that the Cr-rich carbides are partially dissolved and consequently refined by EPT, which could be attributed to the decreased thermodynamic dissolution barriers and accelerated kinetic diffusion of carbon atoms towards dislocation. These results inspire people to develop a novel strategy (CR + EPT) to fully take advantage of CR and tailor the carbides size in bearing steels.

## Introduction

M50 bearing steel has been widely used in the aerospace industry as main shaft bearing in gas-turbine engines due to its excellent elevated temperature performance^1^. In recent years, with the rapid development of aerospace industry, the demand for high properties of aviation bearing steel is constantly increasing for the sake of adapting to the worse working conditions. Therefore, how to improve the mechanical properties of M50 bearing steel has attracted much attention.

As a key shape-forming procedure prior to heat treatment, the cold rolling (CR) technology has been proven to have great potential in improving the strength and toughness of low-alloyed bearing steel owing to grain refinement^2,3^, martensite refinement^4^, bainite refinement^5,6^ and solution strengthening^7^. This raises the speculation that whether the application of CR process will be positive to the comprehensive performance of high-alloyed M50 bearing steel as well. Unfortunately, some results^8,9^ have shown that the mismatch of strain between the hard phases (cementite or alloy carbides) and soft phases (ferrite) could result in the opening up of voids at the carbides/ferrite interfaces during the CR process with a large plastic deformation, which will deteriorate the final mechanical properties of bearing steel. For the high-alloyed M50 steel with poor formability, the voids are more likely to form during the CR process^10^. As a result, the industrial application of CR technology in M50 bearing steel has been limited due to the difficulty in preventing or eliminating the nano-size voids.

Recently, many researches^11–14^ have experimentally proven that the macro cracks can be effectively repaired by electropulsing treatment (EPT) due to the temperature rise and compressive stress. Thus, this crack-healing effect gives us a vision that the combination of CR and EPT may further improve the mechanical properties of M50 bearing steel if the nano-size voids around carbides can be healed as well. However, it is worth noting that the voids resulted from CR process mainly locate around high resistance second-phase (carbides), making the application of EPT to affect the nano-size voids more complicated. Besides, the dislocation entanglement induced by CR may also exert a crucial effect on the void healing.

Therefore, this study presents the effect of EPT on the nano-size voids induced by the CR process, and then the relevant microstructure evolution was characterized and discussed in detail. It is interesting to find that not only the nano-size voids around carbides have been healed extensively, but also the carbides have been refined by the EPT technology.

## Experimental Procedures

M50 bearing steel used in this study with the nominal composition is presented in Table 1. The material was received as spheroidize-annealed bar and an initial microstructure of primary carbides in ferritic matrix. The ring blank used for CR was prepared with the dimension (outer diameter: 54.5 mm; inner diameter: 34.5 mm) and then cold rolled for a total thickness reduction of 50% using a radial ring rolling machine. After the CR tests, the EPT specimens with a size of 16 × 8 × 4 mm were obtained from the CR ring using wire-electrode cutting. The EPT experiments were conducted by a self-made electropulse generator under ambient conditions. The pulsed electric current was applied for a total duration of 160 ms and had a peak current density of 10.7 kA/cm^2^ with a frequency of 50 Hz. During the EPT, the maximum temperature rising of the specimens caused by joule heating was measured to be 532.0 °C by means of an infrared camera (Fotric 226).

**Table 1:** Chemical compositions of M50 bearing steel (wt.%).

C	Cr	Mo	V	Mn	Si	W	Fe
0.8~0.85	4~4.25	4~4.5	0.9~1.1	0.15~0.35	≤0.25	≤0.25	Bal.

In order to characterize the distribution of voids in the matrix before and after EPT, the CR specimens without and with EPT were micro-machined and detected by a Zeiss Auriga field emission scanning electron microscopy (FESEM) equipped with a focused ion beam (FIB) and an energy dispersive spectroscopy (EDS). Based on at least eight FIB-SEM micrographs, the distribution of equivalent diameter of voids was measured using Photoshop and Image-pro Plus software. The quasi *in-situ* observation of the voids was performed in the same field through marking with hardness pits. To investigate the size of carbide particles, the carbides in specimens were extracted by chemical dissolution of the matrix in a modified Berzelius solution^15^ and then analyzed by a Malvern nano particle size analyzer. Additionally, an FEI tecnai F20 transmission electron microscopy (TEM) was employed to observe the microstructure before and after EPT. The specimens for TEM were prepared by mechanically polishing and then electro-polishing in a twin-jet polisher using a solution of 10% perchloric acid and 90% acetic acid.

For observing the effect of EPT on the hardness of ferrite matrix adjacent to carbides, the nano-indentation tests were carried out before and after EPT at peak loads of 1, 2 and 3 mN using a nano-indentation system (NH2, Switzerland). The indentation was applied on the ferrite matrix and confirmed by an atomic force microscope (AFM; DI Nanoscope IV). To investigate the crystal structure information of ferrite, the X-ray diffraction (XRD) data were obtained with a scanning speed of 1°/min on a Rigaku D/MAX-RB diffraction analyser at 12 kW. Furthermore, the differential scanning calorimetry (DSC) experiments were also performed using a PerkinElmer Pyris 1 calorimeter. The specimens for DSC were cut into ϕ 4 mm × 0.5 mm and then heated from ambient temperature to 950 °C at different heating rates of 5, 10, 15, 20 °C/min, respectively.

## Results

**Figure 1:**
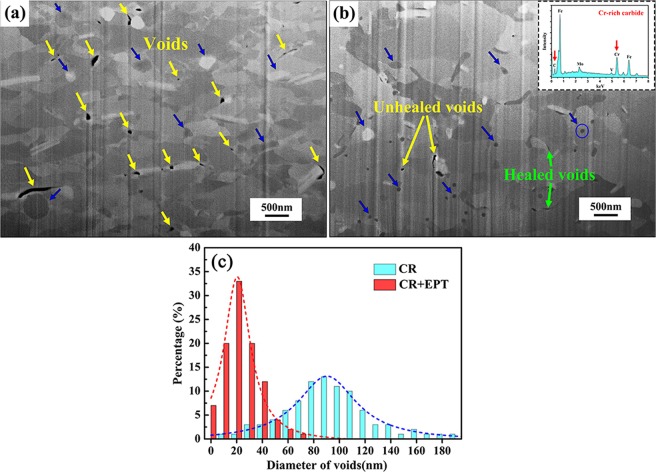
FIB-SEM micrographs collected from the CR specimens (**a**) without and (**b**) with EPT. Insert is the EDS analysis of the nano-size particles (marked with blue circle). (**c**) Distribution of equivalent diameter of voids.

Figure 1 presents the FIB-SEM micrographs of the CR specimens without and with EPT treatment. As indicated in Fig. 1(a), a large number of voids (marked with yellow arrows) less than 1μm have been introduced after the CR process with a 50% thickness reduction. The microstructure of specimens after EPT is shown in Fig. 1(b), where it can be found that some voids have healed (as marked with green arrows) and the number of voids in the matrix have decreased remarkably after EPT. Meanwhile, the distribution of equivalent diameter of voids is further measured as shown in Fig. 1(c). It can be clearly seen that the diameter of voids significantly decreases after EPT, which thus indicates a clear healing effect. In addition, as marked with blue arrows in Fig. 1(a,b), a substantial number of fine carbide particles can be found in the matrix after EPT, while the carbide sizes in the CR specimens are larger than those in the treated specimens. By means of EDS analysis inserted in Fig. 1(b), the nano-size particle (marked with blue circle) is identified to be Cr-rich carbide.

**Figure 2:**
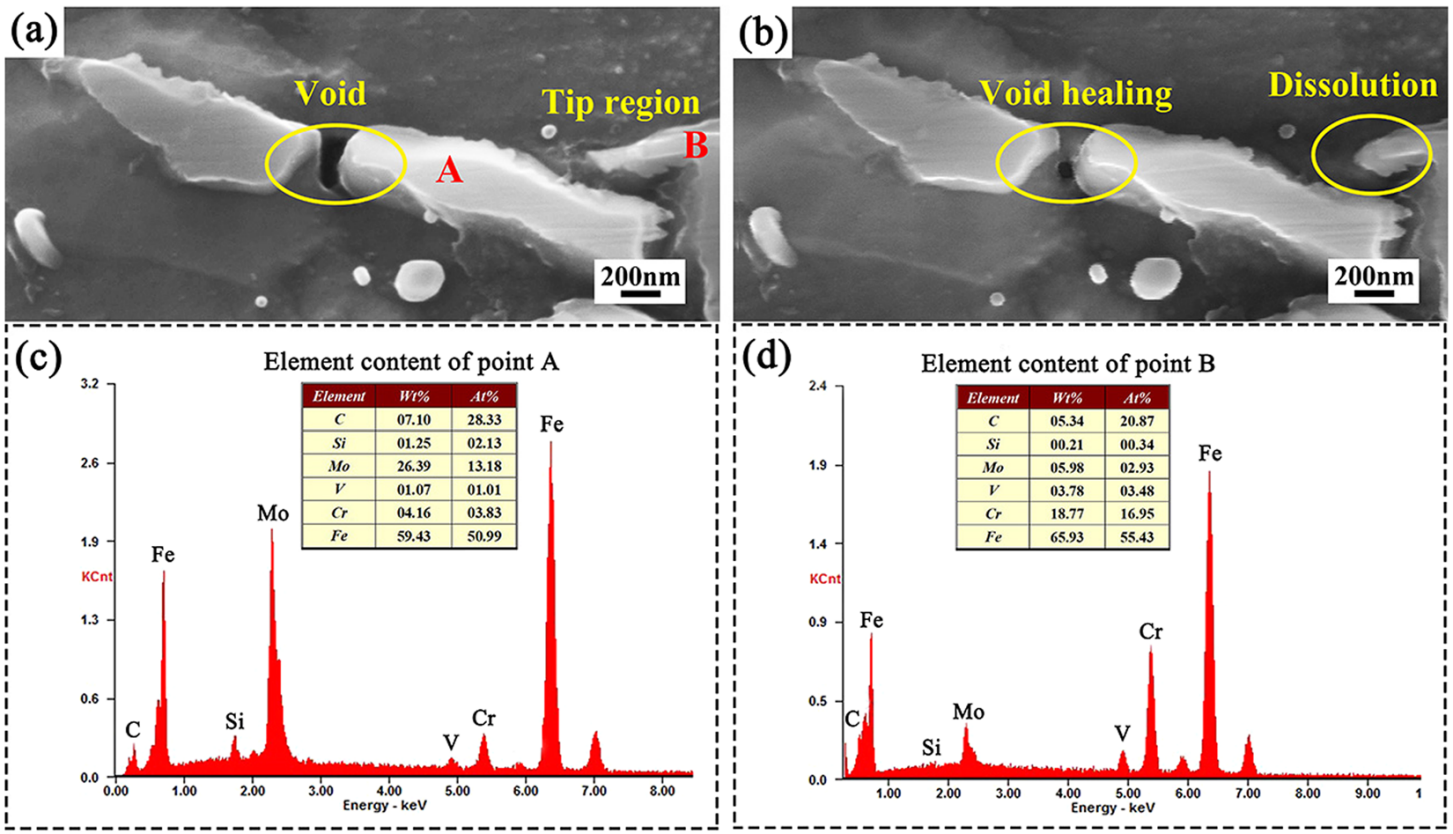
Quasi *in-situ* observation of the microstructure for the CR specimens (**a**) before and (**b**) after EPT; EDS results of (**c**) point A and (**d**) point B.

To further verify the occurrence of voids healing, the quasi *in-situ* observation of the void is performed as shown in Fig. 2. The initial void with long axis of about 260 nm locates between two strip carbides, which may be caused by the movement of carbide fragments during plastic deformation^16^. After EPT, the diameter of void decreases to about 80 nm, thereby validating the contribution of EPT to the healing of the voids around carbides. It is also found that the tip region of deformed carbide has been partially disappeared after EPT as circled in Fig. 2(b), which may result from the localized dissolution of carbides. Furthermore, the EDS results of point A and B are shown in Fig. 2(c,d). It can be seen that there is larger amount of Mo element existing in the broken strip carbide (marked with A), whereas the partially dissolved carbide is rich in Cr element (marked with B). The results, together with the chemical analysis in Fig. 1, prove that the Cr-rich carbides have lower stability under EPT.

**Figure 3:**
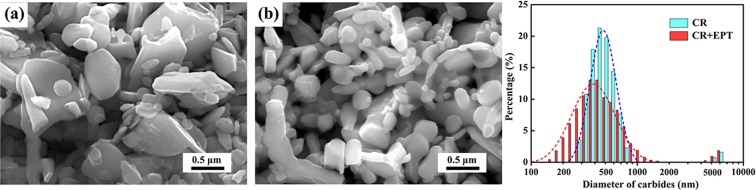
SEM observation of the extracted carbides from the CR specimens (**a**) without and (**b**) with EPT, and (**c**) diameter distribution of extracted carbides.

As shown in Fig. 3, the carbides in the CR specimens without and with EPT are extracted and then observed by SEM. It clearly shows that the extracted carbides of the EPT specimen is finer than that of specimen without EPT. According to the analysis of particle size of carbides, the average diameter of carbides decreases from 495 nm to 412 nm after EPT. Furthermore, the particle size distribution of extracted carbides (Fig. 3(c)**)** indicates that the proportion of carbides with a diameter less than 200 nm is significantly increased after EPT, which therefore coincides with the observation in Fig. 1(b).

**Figure 4:**
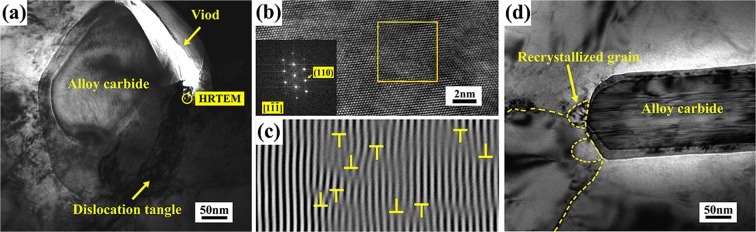
TEM observation of the microstructure from the CR specimens (**a**) without and (**d**) with EPT, (**b**,**c**) HRTEM observation of the dislocation tangle around carbide in the CR specimens.

Figure 4 shows the TEM observation of the microstructure for the CR specimens without and with EPT. For the CR specimens without EPT, the void adjacent to carbide can be found as shown in Fig. 4(a). The HRTEM observation in Fig. 4(b,c) indicates that high dislocation tangle has entangled in the ferrite matrix around carbides for the CR specimens. This result may lie in the fact that the carbides could act as the strong obstacles to inhibit dislocation motion during the CR process. However, the significant recovery of entangled dislocations occurs within a short time of EPT (Fig. 4(d)), which could be attributed to the increased mobility of dislocation by the effect of electron wind force^17^. In addition, it is also showed that fine recrystallization grains formed around the carbide, indicating the occurrence of rapid recrystallization during the EPT.

**Figure 5:**
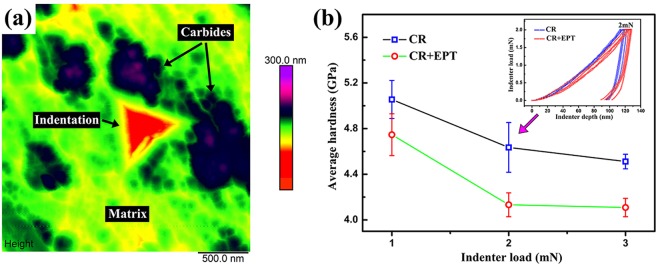
(**a**) AFM image of the nano-indentation for the specimen with EPT at peak load of 2 mN; (**b**) Average hardness of the ferrite matrix for the CR specimens without EPT and with EPT. The insert is load-displacement curves at peak loads of 2 mN.

The nano-indentation tests are carried out to investigate the hardness of ferrite without and with EPT. As shown in Fig. 5(a), the indentation has been confirmed to be applied on the ferrite matrix and then observed by AFM. The statistical results in Fig. 3(b) show that the average hardness of the ferrite matrix distinctly decreases after EPT regardless of the indenter load, which may be attributed to the recovery of dislocation and occurrence of rapid recrystallization during the EPT (Fig. 4(d))^18^.

## Discussion

In general, the void healing effect induced by EPT can be explained by the inhomogeneous temperature rise caused by the difference of electric resistance between voids and matrix. This inhomogeneous temperature rise can lead to inhomogeneous thermal expansion, so that the voids will be subjected to a strong thermal compressive stress and then consequently healed^19^. In this work, for the tip region of deformed carbide, the temperature rise around the carbide will be higher because of the detour effect of EPT. As a result, the thermal compressive stress will be greater around the tip region of carbides, which may be more conducive to the healing of the voids around the deformed carbides. Additionally, the dislocation motion around carbides may be another important factor to facilitate the voids healing process. The drift electrons can exert a push on dislocations when high density electric pulses are passing through the CR specimen. Under this force, the rearrangement and annihilation of the dislocations around carbides (Fig. 4(d)) will increase the diffusion velocity of atoms^20^. Meanwhile, it has been experimentally proved that the voids healing process is controlled by lattice diffusion (transportation of atoms and vacancies)^21^. Consequently, the generation of thermal compressive stress and acceleration of atoms diffusion will together contribute to the voids healing process of cold rolled M50 bearing steel during EPT.

Previous studies^22,23^ have shown that the EPT can lead to the dissolution of second-phase below the thermodynamic dissolution temperature within a very short time. Due to the thermal effect of EPT, the real temperature of the treated specimens may have approached or exceeded the critical thermodynamic dissolution temperature. In the meantime, with the enhance of the athermal effect of EPT, the thermodynamic barrier decreases and then the dissolution behavior of the second phase occurs. In this work, considering that the temperature rise induced by joule heating only reached 532 °C, a temperature far away from the critical dissolution temperature of carbides in M50 bearing steel^24^, the complete dissolution of carbides will not be expected. However, the refinement behavior of carbides in the matrix are surprisingly discovered (Fig. 3), which have been rarely reported so far and will be further discussed.

The change of free energy (${\rm{\Delta }}{{G}_{dis}}^{EPT}$) for the second-phase dissolution process under EPT can be simplified as^25^:

$${\rm{\Delta }}{{G}_{dis}}^{EPT}={\rm{\Delta }}{{G}_{dis}}^{{\rm{o}}}+{\rm{\Delta }}{{G}_{dis}}^{e}$$

where ${\rm{\Delta }}{{G}_{dis}}^{{\rm{o}}}$ is the free energy change for carbide dissolution in a current-free system, ${\rm{\Delta }}{{G}_{dis}}^{e}=\frac{{\sigma }_{{matrix}}-{\sigma }_{{carbide}}}{2{\sigma }_{{matrix}}+{\sigma }_{{carbide}}}k{j}^{2}V$ is the energy change due to the passage of pulse current through the specimens^22^. *k* is the geometric factor, *j* is the current density, and *V* is the volume. The higher conductivity of ferrite matrix (${\sigma }_{{matrix}} > {\sigma }_{{carbide}}$) results in ${\rm{\Delta }}{{G}_{dis}}^{e} > 0$, which will cause the carbides to be unstable and facilitate the dissolution of carbides into the ferrite matrix as an driving force. Additionally, for the contribution of ${\rm{\Delta }}{{G}_{dis}}^{{\rm{o}}}$, the interfacial energy contribution plays an important role and can be approached by ${\rm{\Delta }}{{G}_{{dis}}}^{{inter}}=\gamma {V}_{m}\frac{dA}{d{V}_{{carbide}}}$^26^. Where *y*, *V*_*m*_, *A* and *V*_*carbide*_ are the specific interfacial energy, the molar volume of carbide, the surface area between carbide and matrix, and the carbide volume, respectively. It can be inferred that the increased surface to volume ratio of matrix/carbide (especially at the tip region of the deformed carbide) will enhance the interfacial energy contribution to the thermodynamically carbide dissolution. Accordingly, the partial dissolution (Fig. 2) potentially results from the lower thermodynamic dissolution barrier for the deformed carbide with a higher surface to volume ratio of matrix/carbide.

Figure 4(a) shows that high density dislocation is adjacent to a ferrite/carbide interface, which are likely to draw carbon atoms from the carbide due to the higher binding energy of carbon atoms with dislocation^27^. Once the sink of carbon atoms in the ferrite become supersaturated, partial dissolution of carbides will take place. This phenomenon generally occurs during the process of strain aging^28,29^. However, when kinetic diffusion of carbon atoms is accelerated by the pulsed electric, the localized carbide dissolution would occur within a short time. Meanwhile, it should be noted that the detour effect^30^ of electric current results in a higher joule heating adjacent to voids and the tip region of carbide than the overall estimated temperature (532 °C), which will lead the local temperature of carbides to be close to the thermodynamic dissolution temperature. Since the thermodynamic dissolution temperature of Cr-rich carbides is lowest^23^, the partial dissolution and refinement could be easier to achieve. Conclusively, according to the chemical composition and morphology observation of the refined carbides, it can be inferred that the Cr-rich carbide with a higher surface to volume ratio could be partially dissolved and refined by EPT (especially at the tip region of the deformed Cr-rich carbide).

**Figure 6:**
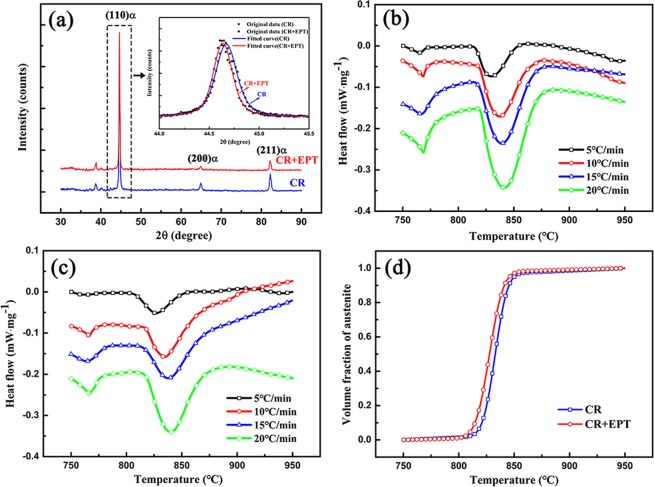
(**a**) The XRD patterns of the CR specimens without and with EPT; the DSC curves of the CR samples (**b**) without and (**c**) with EPT at different heating rates; (**d**) The volume fraction of austenite formed as a function of temperature at 5 °C/min.

In order to further verify the partial dissolution behavior of carbides, the XRD patterns for the CR specimens with and without EPT are obtained, as shown in Fig. 6(a). According to the Gaussian fitted curves of (110)α diffraction peak (as insert in Fig. 6(a)), the values of FWHM can be measured (Table 2) and shows a distinct decrease after EPT, which therefore verifies the dislocation recovery as observed in Fig. 4. Moreover, it can be clearly seen that the ferrite diffraction peak shifts to smaller angle after EPT. This indicates that the lattice parameter of α-Fe increases after EPT. The carbon content in the ferrite can be estimated based on the relationship between the lattice parameter and carbon constant of α-Fe given by Fasiska and Wagenblast^31^ as follow: $${a}_{\alpha }(nm)=(0.28664\pm 0.0001)\,$$$$+\,(0.84\pm 0.08)\times {10}^{-3}\cdot {[C]}_{\alpha }$$. where *a*_*α*_ is the lattice parameter of α-Fe, and [*C*]_*α*_ is the carbon content in α-Fe (at.%). Based on the measured lattice parameters, the carbon content in α-Fe before and after EPT is calculated and listed in Table 2, where it can be seen that the carbon content in α-Fe increases substantially after EPT. Obviously, compared to the carbon content in equilibrium ferrite (the solid solubility of carbon in ferrite at 727 °C is 0.0218 wt.%^32^), the determined carbon content in ferrite after EPT is supersaturated. This result clearly confirms the migration of carbon from carbides to ferrite and partial dissolution of carbides during EPT.

**Table 2:** Crystal structure information of ferrite and activation energy of α → γ without and with EPT determined by X-ray diffraction and differential scanning calorimetry, respectively.

Specimen	FWHM (110α)	*a*_*α*_ (nm)	[*C*]_*α*_ (at.%)	[*C*]_*α*_ (wt.%)	Q_α→γ_ (kJ·mol^−1^)
CR	0.28258	0.28670	0.0714	0.0153	1120
CR + EPT	0.25351	0.28691	0.3214	0.0689	1036

Furthermore, the carbon-supersaturated ferrite results in a decrease of the carbon chemical potential between the ferrite and carbides, which will facilitate the transformation of ferrite to austenite^33^. Figure 6 (b,c) illustrates the DSC curves of the CR samples without and with EPT at different heating rates. Obviously, the peak transformation temperature of α → γ increases with the increase of heating rate. Based on the Kissinger method^34^, the activation energy for the transformation from ferrite to austenite (Q_α→γ_) is obtained in Table 2, which indicates a distinct decrease of Q_α→γ_ after EPT. Meanwhile, the volume fraction of austenite formed as a function of temperature at 5 °C/min is plotted in Fig. 6(d), which demonstrates that the transformation of α → γ shifts to lower temperature. Therefore, the EPT not only leads to partial dissolution of carbides but also accelerates the transformation from ferrite to austenite during subsequent heating by increasing the carbon solid-solute in ferrite.

## Conclusion

In summary, through utilizing the designed EPT processing technology, the extensive voids healing and carbide refinement can be realized within a short time (millisecond level). The generation of thermal compressive stress and acceleration of atoms diffusion will together contribute to voids healing process of cold rolled M50 bearing steel. As well, for the deformed Cr-rich carbide with a higher surface to volume ratio of matrix/carbide, the partial dissolution and refinement of carbides could easily occur due to the decreased thermodynamic dissolution barriers and accelerated kinetic diffusion of carbon atoms towards dislocations during EPT. Moreover, this partial dissolution behavior will result in carbon enrichment in ferrite and accelerate the transformation from ferrite to austenite during subsequent heating.

The novel phenomenon provides a new pathway for fully eliminating the deteriorating effect of voids on mechanical properties and maximizing the advantages of CR in bearing steel. Meanwhile, it is of great engineering significance to tailor the carbides size in bearing steels using the efficient and energy-saving EPT processing technology, in the context of cold rolled bearing steel.
